# Unexpected Localization of AGP Epitopes in the Plastids of *Aldrovanda vesiculosa*: New Questions About the Distribution of These Epitopes in Angiosperms

**DOI:** 10.3390/ijms27198452

**Published:** 2026-09-22

**Authors:** Bartosz J. Płachno, Małgorzata Kapusta, Marcin Feldo, Piotr Stolarczyk, Piotr Świątek, Agata Leszczuk

**Affiliations:** 1 Institute of Botany, Faculty of Biology, Jagiellonian University, 9 Gronostajowa St., 30-387 Kraków, Poland; 2 Bioimaging Laboratory, Faculty of Biology, University of Gdańsk, 59 Wita Stwosza St., 80-308 Gdańsk, Poland; 3 Department of Vascular Surgery and Angiology, Medical University of Lublin, 16 Staszica St., 20-081 Lublin, Poland; 4 Department of Botany, Physiology and Plant Protection, Faculty of Biotechnology and Horticulture, University of Agriculture in Kraków, 29 Listopada 54 Ave., 31-425 Kraków, Poland; 5 Institute of Biology, Biotechnology and Environmental Protection, Faculty of Natural Sciences, University of Silesia in Katowice, 9 Bankowa St., 40-007 Katowice, Poland; 6 Institute of Agrophysics, Polish Academy of Sciences, Department of Soil Physicochemistry and Remediation, Doświadczalna 4, 20-290 Lublin, Poland

**Keywords:** *Aldrovanda*, arabinogalactan proteins, aquatic plants, carnivorous plants, chloroplasts, Droseraceae, plastids, starch, turions, waterwheel plant

## Abstract

Arabinogalactan proteins (AGPs) are the glycoproteins that are characteristic of plant cells (except for some algae) and are essential for cell function (cell–cell communication, cell adhesion, cell expansion, cell-wall organization, and signaling) but also for the development of roots, shoots, leaves, flowers, and reproductive structures. They occur inside and on the surface of plant cells, but mainly at the interface between the plasma membrane and the cell wall. Reports on the occurrence of arabinogalactan proteins in plastids are very rare and unclear. Our goal was to determine whether AGP-reactive carbohydrate epitopes are associated with plastids and starch in turion cells of *Aldrovanda vesiculosa* L. To do this, the localization of the AGP-reactive carbohydrate epitopes was assessed using immunolabeling and confocal and electron microscopy. Abundant signals for the antibodies LM2 and JIM8 against the AGP epitopes were detected in plastids. The LM2 signals were particularly frequent in starch grains. Also, electron microscopy confirmed the presence of AGP-reactive carbohydrate epitopes in plastids. These findings provide further evidence for the unusual intracellular localization of AGP-reactive carbohydrate epitopes in plastids and highlight starch grains as a target for future studies aimed at determining the molecular identity and biological significance of these epitopes.

## 1. Introduction

Arabinogalactan proteins (AGPs) are the glycoproteins featuring a PAST-rich (proline, alanine, serine, threonine) protein domain with repetitive dipeptides, covalently linked type II arabinogalactans, an N-terminal secretion signal, and a C-terminal glycosylphosphatidylinositol (GPI) anchor for membrane attachment [1,2,3,4,5]. They have the ability to bind the β-Yariv reagent. It recognizes the β-1,3-galactan backbone of their arabinogalactan chains [6,7,8,9]. AGPs are widespread among angiosperms [2], gymnosperms [10,11], pteridophytes [12,13,14,15], and bryophytes [16,17,18,19,20,21].

They are found in various types of plant organs, tissues, and cells [2], including those involved in sexual reproduction (e.g., [22,23,24,25,26]). In some plant species, AGPs may be used as molecular markers of sexual reproduction (e.g., [27,28]). They play diverse but crucial roles both in the cells themselves and in the development of organs and embryos, including cell–cell communication, cell adhesion, cell expansion, cell-wall organization, and signaling [29,30,31,32,33,34].

The distribution of AGPs varies. They mostly occur in the plasma membrane–cell wall interface [35]. GPI-anchored AGPs are localized in the plasma membrane and thus provide a physical and potentially functional continuum between the plasma membrane and extracellular matrix, including the cell wall [2,36,37]. They were found in the intercellular spaces as well as in soluble exudates (e.g., root or seed mucilage) secreted by plants [38,39,40,41]. However, AGPs also occur in the cytosol, and they are associated with various endomembranes of plant cells. Samaj et al. [42] and other authors [40,43,44,45,46] found an association between AGPs and endomembranes, including the ER, tonoplasts, vesicles, Golgi apparatus, and multivesicular bodies. This is logical because AGPs are synthesized via the secretory pathway as hydroxyproline-rich proteins and undergo extensive post-translational processing in the ER and the Golgi apparatus. Following translocation into the ER, the N-terminal signal peptide is removed, proline residues are hydroxylated, and a GPI anchor is added to the C-terminus of the AGP. Glycosylation is initiated by the addition of galactose to hydroxyproline residues and is subsequently extended by a series of glycosyltransferases that assemble the characteristic type II arabinogalactan chains, comprising β-(1 → 3)-galactan backbones with β-(1 → 6)-galactan branches that can be further substituted with arabinose, glucuronic acid, fucose, and other sugars [1,30,46]. The extensive glycan assembly occurs predominantly during passage through the Golgi, where the molecular structure of individual AGPs is further modified by tissue- and protein-specific glycosyltransferase activities. Following completion of glycosylation, AGPs are transported by secretory vesicles to the plasma membrane, where GPI-anchored AGPs are exposed at the extracellular surface and may subsequently be released into the cell wall or apoplast following cleavage of the GPI anchor [1,30,46].

In many studies, authors who describe the occurrence of AGPs in plant cells use the term “in cytoplasm” as a shorthand, implying a cytoplasmic endomembrane system (e.g., [47,48,49,50,51,52,53,54]). Sometimes, even when the authors use the immunogold technique for epitope localization, they can only determine that AGPs occur in the “cytoplasm,” and this is associated with poor contrast or poor fixation, which limits antibody reactivity.

In our previous study, we analyzed turions of various aquatic species for the occurrence of AGP-reactive carbohydrate epitopes [55]. These turions are also rich in reserve materials (proteins, lipids, fatty acids), including starch [56,57,58]. An unexpected and surprising result was that we found AGP-reactive carbohydrate epitopes localized in the plastids of turions of *Aldrovanda vesiculosa* L. We searched the literature for the occurrence of AGPs in plastids and found only a few papers. Costa et al. [59] observed that JIM8, LM2, and JIM13 epitopes occurred on the outer surfaces or even within starch grains of multiple tissues of *Trithuria submersa* Hook.f. Costa et al. [60] found that the MAC207 epitope surrounded starch-rich vesicles in anther cells. However, this study does not provide a clear picture of the precise localization of AGPs within plastids. Both studies were performed using light microscopy. A very interesting observation was the finding by Leszczuk and Szczuka [61] that AGPs are in the megaspore of *Fragaria* × *ananassa* (Duch.).

The selection of *A. vesiculosa* was not intended to imply that this species is representative of the more than 600 known species of carnivorous plants. Rather, it was specifically motivated by our previous study, in which we unexpectedly detected AGP epitopes within plastids of *A. vesiculosa* turion cells [55]. This observation was particularly intriguing because AGPs are generally associated with the plasma membrane–cell wall continuum and, during their biosynthesis, pass through the conventional secretory pathway involving the endoplasmic reticulum and Golgi apparatus. The apparent presence of AGP epitopes inside plastids, therefore, represented an unusual localization that warranted independent confirmation using improved histological preparation and higher-resolution microscopy. *A. vesiculosa* was consequently selected as a follow-up experimental system because it provided a previously identified and clearly defined biological context in which this unexpected phenomenon could be investigated in greater detail. Our goal was to reanalyze plastids from *Aldrovanda* turion cells to confirm the occurrence of AGP-reactive carbohydrate epitopes in plastids and their association with starch. Importantly, our aim is not to claim that AGP-reactive carbohydrate epitopes are universally localized to plastids in carnivorous plants, nor to infer a specific function from localization alone. Rather, the significance of the present study is that it provides additional microscopic evidence for an unusual intracellular localization of AGP-reactive carbohydrate epitopes and documents their spatial relationship with starch-containing plastids in a well-defined storage organ.

We hypothesized that AGP-reactive epitopes are specifically associated with plastids in *Aldrovanda vesiculosa* turion cells and that some of these epitopes are also associated with starch grains.

## 2. Results

### 2.1. Plastid Distribution

The leaves forming the turions were green (Figure 1A), and the plastids contained chlorophyll. Chloroplasts occurred in both epidermal and aerenchyma cells. In the aerenchyma cells, plastids formed clusters around the cell nuclei (Figure 1B,C). The plastids contained starch grains (a single plastid could contain several grains), which were clearly visible following the Periodic Acid–Schiff (PAS) reaction, as a result of which the starch grains turned pink–purple (Figure 1D).

### 2.2. Distribution of AGP-Reactive Carbohydrate Epitope—Detection Using the Antibody LM2

LM2 signals were well observed in the starch grains in plastids (Figure 2A–D). The gold particles were present in the plastids (Figure 3A–D). Analysis of the presence of gold particles in starch grains was not possible due to resin cracking at these sites.

### 2.3. Distribution of AGP-Reactive Carbohydrate Epitope—Detection Using the Antibody JIM8

JIM8 signals were observed in starch grains within plastids and near the cell walls (Figure 4A,B). The gold particles were present in the plastids’ stroma (Figure 5A–D) and starch grains (Figure 5C,D).

## 3. Discussion

### 3.1. AGP-Reactive Epitopes Are Associated with Plastids of Aldrovanda vesiculosa

The present study provides evidence that LM2- and JIM8-reactive AGP epitopes are present within the plastids of *Aldrovanda vesiculosa* turion cells. This finding extends the previously reported intracellular distribution of AGP-reactive epitopes [55]. AGPs are generally associated with the plasma membrane and extracellular matrix, where they participate in interactions between the cell surface and the cell wall. However, AGP epitopes have also been detected in intracellular endomembrane compartments, including the endoplasmic reticulum, Golgi apparatus, vesicles, and tonoplast, consistent with their biosynthesis and trafficking through the secretory pathway [30,42]. The present observations therefore add plastids to the range of intracellular compartments in which AGP-reactive epitopes have been detected.

The two microscopic approaches used here provide complementary evidence for this localization. Confocal microscopy revealed LM2- and JIM8-reactive fluorescence within plastids, with particularly prominent LM2 fluorescence associated with starch-containing regions. Because of the spatial resolution of confocal microscopy, however, fluorescence cannot reliably distinguish between the starch grain, the surrounding stroma, and the interface between these compartments. Immunogold electron microscopy provided higher spatial resolution and confirmed the occurrence of both LM2- and JIM8-reactive epitopes within plastids. Gold particles were observed in the plastid stroma and in regions corresponding to starch grains. The lower density of immunogold labeling compared with the fluorescence signal does not constitute a contradiction between the methods. Immunogold labeling is strongly influenced by fixation, resin embedding, epitope preservation, and accessibility of antibody-binding sites. Consequently, the density of gold particles cannot be directly compared with the intensity of a confocal fluorescence signal.

These observations support the localization hypothesis addressed by the present study, namely that AGP-reactive epitopes are genuinely associated with plastids of *A. vesiculosa* turion cells and that some of these epitopes are associated with starch-containing regions. The ultrastructural observations are particularly important because they reduce the possibility that the fluorescence observed in plastids results solely from optical overlap with neighboring AGP-containing structures. Nevertheless, the data demonstrate the localization of the epitopes recognized by LM2 and JIM8 rather than establishing the presence of complete AGP molecules in plastids.

### 3.2. Association of AGP-Reactive Epitopes with Starch-Containing Plastids

A notable feature of the present observations is the repeated association of AGP-reactive epitopes with starch-containing plastids. LM2 fluorescence was particularly prominent in starch grains, whereas immunogold microscopy confirmed LM2- and JIM8-reactive epitopes within plastids. Similar observations have been reported in other angiosperms. Costa et al. [59] detected JIM8, JIM13, and LM2 epitopes associated with starch grains in reproductive tissues of *Trithuria submersa*. Leszczuk and Szczuka [61] also reported AGP epitopes associated with starch grains in *Fragaria × ananassa*. Thus, the occurrence of AGP-reactive epitopes in plastids and starch grains has now been observed in several phylogenetically distant angiosperms.

The repeated observation of this association is noteworthy, but its biological significance remains unresolved. The present data establish spatial association rather than a functional relationship between AGP-reactive epitopes and starch. In particular, they do not demonstrate that these epitopes participate in starch synthesis, degradation, accumulation, or mobilization. The association may therefore represent a feature of the intracellular distribution of particular carbohydrate epitopes rather than evidence for a direct role of AGPs in starch metabolism.

### 3.3. Possible Origin of AGP-Reactive Epitopes in Plastids

The detection of AGP-reactive epitopes in plastids raises the question of their molecular origin. Classical AGPs are synthesized through the secretory pathway. They commonly contain N-terminal signal sequences directing the nascent protein into the secretory system, while many also possess C-terminal signals associated with GPI anchoring. Their maturation involves proline hydroxylation and extensive O-glycosylation, processes associated principally with the endoplasmic reticulum and Golgi apparatus [1,3,5,46]. The present observations, therefore, do not provide evidence that complete, canonical AGPs are synthesized within plastids.

One possible explanation is that AGP-containing material or AGP-derived glycan structures reach plastids after processing in the secretory system. Intracellular AGP epitopes have previously been detected in several endomembrane compartments, supporting the view that AGP-related material can occur along the secretory pathway [40,42]. For example, immunogold studies in tobacco BY-2 cells detected AGP epitopes in the endoplasmic reticulum, Golgi apparatus, and vesicles [40]. An alternative possibility is that the antibodies recognize AGP-related glycoconjugates or glycan fragments rather than intact AGP molecules. This distinction is particularly important because LM2 and JIM8 recognize carbohydrate epitopes and do not identify the complete protein carrying the epitope. Accordingly, the present immunolocalization results establish the presence of the recognized carbohydrate structures but cannot determine the molecular identity or biosynthetic origin of the labeled material.

### 3.4. Biological Significance and Limitations of the Present Observations

Turions of *A. vesiculosa* are dormant organs that accumulate storage compounds, including starch, which can subsequently support renewed growth after dormancy [57,62,63]. The association of AGP-reactive epitopes with starch-containing plastids is therefore particularly interesting in this physiological context. AGPs have established roles in several aspects of plant development and cell-surface organization [3,5,31], and glucuronic acid-containing arabinogalactan polysaccharides can interact with Ca^2+^ [32]. These observations provide a biological context in which the present localization could be investigated further.

However, the present experiments do not demonstrate a functional role for AGP-reactive epitopes in calcium homeostasis, starch metabolism, or plastid function. Such interpretations would require direct measurements of AGP-associated molecules, calcium distribution, starch metabolism, and the relevant physiological processes. The particularly strong LM2 signal in starch grains, therefore, represents a useful observation for generating hypotheses, but it should not be interpreted as evidence of a specific biochemical function.

An important strength of the present study is the use of two complementary microscopic approaches. Confocal microscopy provides information on the overall spatial distribution of the immunoreactive signal, whereas immunogold electron microscopy enables assessment of its subcellular localization at substantially higher resolution. The agreement between the two approaches strengthens the conclusion that LM2- and JIM8-reactive epitopes are associated with plastids. At the same time, the different methodological characteristics of the two techniques explain why the intensity and apparent abundance of labeling cannot be compared directly.

Finally, the observations should be described specifically in terms of AGP-reactive epitopes. AGPs comprise a structurally heterogeneous group of highly glycosylated proteins, and individual antibodies recognize specific carbohydrate motifs whose distribution may vary across tissues and developmental stages. The detection of LM2- and JIM8-reactive epitopes in *A. vesiculosa* plastids, therefore, does not demonstrate that all AGPs occur in plastids. Nor does it establish whether the labeled structures represent intact AGPs, processed AGP-derived glycans, or other glycoconjugates carrying similar epitopes. Taken together, the present results provide evidence that LM2- and JIM8-reactive AGP epitopes occur within plastids of *A. vesiculosa* turion cells and that these epitopes can be associated with starch-containing regions. The combination of confocal and immunogold microscopy provides complementary evidence for this localization, while also demonstrating the importance of distinguishing epitope localization from identification of complete AGP molecules. The biological significance and molecular origin of the plastid-associated epitopes remain unresolved. Future studies combining biochemical isolation, proteomic identification, and ultrastructural co-localization with plastid envelope and starch-associated markers will be required to determine the nature of the labeled molecules and to establish whether their association with plastids and starch has a specific biological function.

## 4. Materials and Methods

### 4.1. Plant Material

Mature turions of *Aldrovanda vesiculosa* L. (Droseraceae) were collected from a private collection in Poland. The plants were grown in a pot equipped with a perforated insert. Beneath the insert, dead *Carex* sp. leaves and approximately half a handful of bivalve shells were placed. The water volume was approximately 15 L. The setup was maintained outdoors at ground level in a partially shaded area that received full sunlight at certain times of the day. During winter, the temperature was maintained between 2 and 5 °C. The water and *Carex* material were replaced approximately once a year. Turions were fixed in January 2026. At least four turions (four plants, one turion per plant) have been fixed and processed.

### 4.2. Light Microscopy

Turions were photographed using an Emspira 3 Digital Microscope (Leica Microsystem, PIK Instruments—Local Authorized Partner, Laszczki, Poland). Semi-thin sections were prepared for light microscopy and stained for general histology using aqueous methylene blue/azure II (MB/AII) for 1–2 min. A histochemical procedure on turion sections using the Periodic Acid–Schiff (PAS) reaction was performed to detect starch grains. The images were captured using a Nikon Eclipse E400 microscope (Nikon Corporation, Tokyo, Japan).

### 4.3. Immunochemical Analysis

The turions were cut into small fragments and were fixed in 8% (*w*/*v*) paraformaldehyde (Sigma-Aldrich Sp. z o.o., Poznań, Poland) and 0.25% (*v*/*v*) glutaraldehyde (Sigma-Aldrich Sp.z o.o., Poznań, Poland) in PIPES buffer (Sigma-Aldrich Sp. z o.o., Poznań, Poland). The fixed samples were embedded in LR White Resin (Polysciences Europe GmbH, Hirschberg an der Bergstrasse, Germany) and sectioned at a thickness of 0.7 μm using an ultramicrotome (Ultracut UCT, Leica Microsystems, Wetzlar, Germany). The sections were rehydrated, blocked with 1% bovine serum albumin (BSA, Sigma-Aldrich, Poznań, Poland) in PBS, and incubated overnight at 4 °C with the primary antibodies against arabinogalactans (JIM8 and LM2). The goat anti-rat secondary antibody was used. All primary antibodies were purchased from Plant Probes (Leeds, UK) or Kerafast (Newark, CA, USA), and the FITC-conjugated goat anti-rat IgG secondary antibody was purchased from Abcam (Cambridge, UK). The samples were then cover-slipped using a Mowiol mounting medium: a mixture of Mowiol^®^ 4–88 (Sigma-Aldrich Sp. z o.o., Poznań, Poland), glycerol (for fluorescence microscopy; Merck, Warsaw, Poland), and 2.5% (*w*/*v*) DABCO (Carl Roth, Karlsruhe, Germany). They were observed using a STELLARIS 5 WLL confocal microscope with the LIGHTNING deconvolution module (Leica Microsystems, Wetzlar, Germany). Negative controls were created by omitting the primary antibody step, which caused no fluorescence (Figure S1).

### 4.4. Immunogold Labeling of AGP Epitopes

Immunogold labeling was performed as described by Płachno et al. [64]. The cells were visualized using an FEI Tecnai Spirit BioTWIN transmission electron microscope (Thermo Fisher Scientific, Waltham, MA, USA) and also a JEM-100SX transmission electron microscope (JEOL, Tokyo, Japan). Negative controls for all immunogold were created by omitting the primary antibody step (Figure S1).

## 5. Conclusions

We demonstrated that LM2- and JIM8-reactive AGP epitopes occur within the plastids of *Aldrovanda vesiculosa* turion cells, with particularly pronounced LM2 labeling associated with starch grains (as shown in the confocal microscopy results). The combination of confocal microscopy and immunogold electron microscopy provides evidence that this localization is not limited to the cell wall or plasma membrane and supports the existence of AGP-reactive material within the plastid compartment. The findings are consistent with the earlier observation of AGP epitopes associated with starch grains in *Trithuria* and *Fragaria*, suggesting that plastid-associated AGP epitopes may occur more widely in angiosperms than previously recognized. However, the present results do not establish whether intact AGPs are transported to plastids, whether AGP-derived molecules reach this compartment, or whether the detected epitopes are associated with previously uncharacterized plastidial glycoconjugates. Future studies combining immunogold co-localization, biochemical isolation of plastid fractions, mass spectrometry, and analysis of AGP-associated glycans will be required to resolve the molecular identity and biological function of these plastid-associated AGP epitopes.

## Figures and Tables

**Figure 1:**
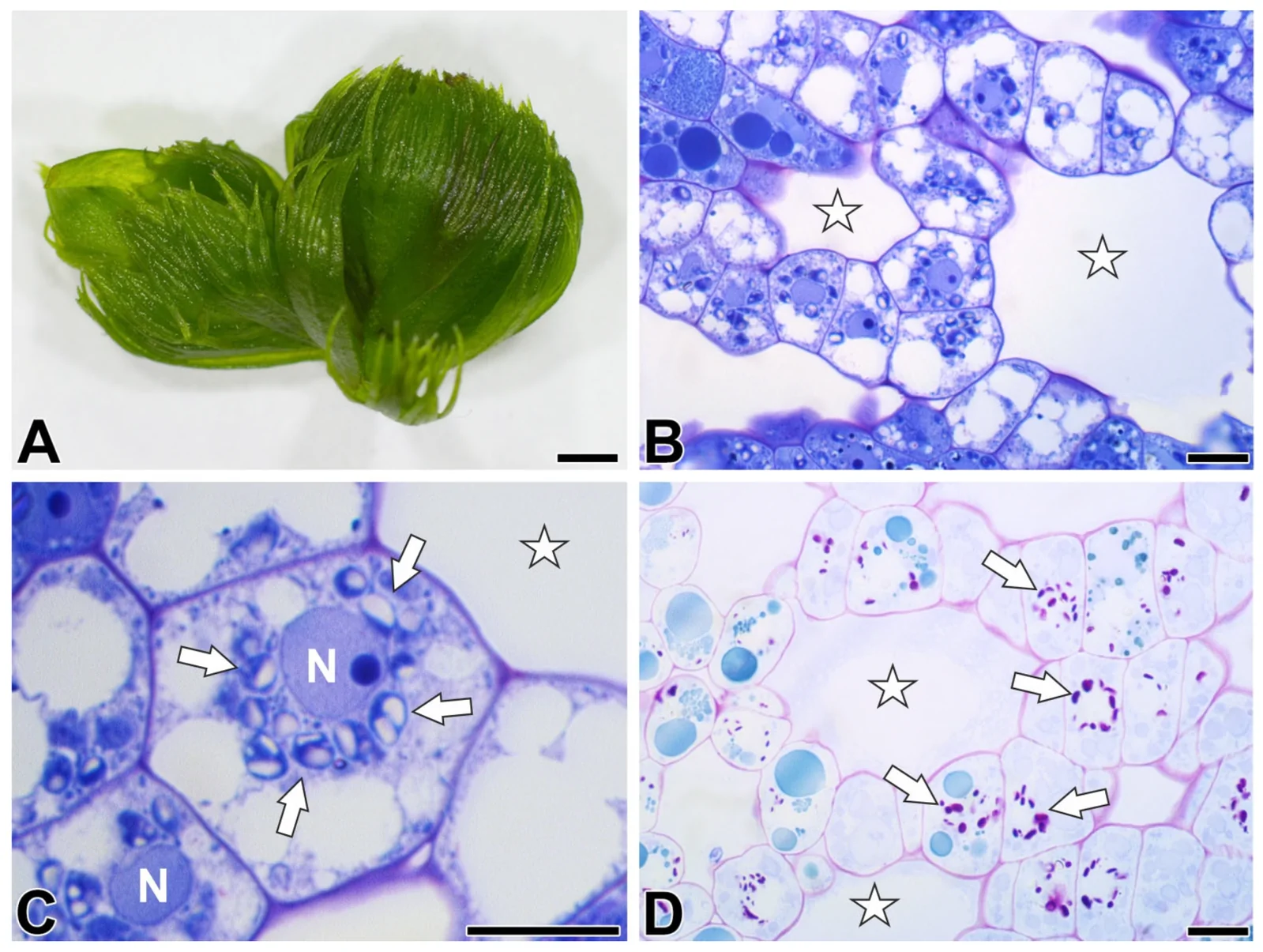
Plastid distribution in turion of *Aldrovanda vesiculosa,* light microscopy; (**A**) Turion of *Aldrovanda vesiculosa*. Bar = 1 mm. (**B**,**C**) Semi-thin sections of turion leaves, stained with aqueous methylene blue/azure II. Note that in aerenchyma cells, plastids (arrows), which contain starch grains, surround the cell nuclei (N). Air duct, star; bar, 10 μm. (**D**) A semi-thin section of turion leaf, note positive PAS reaction in case of starch grains, which turned a vivid pink–purple color (arrows). Air duct, star; bar, 10 μm.

**Figure 2:**
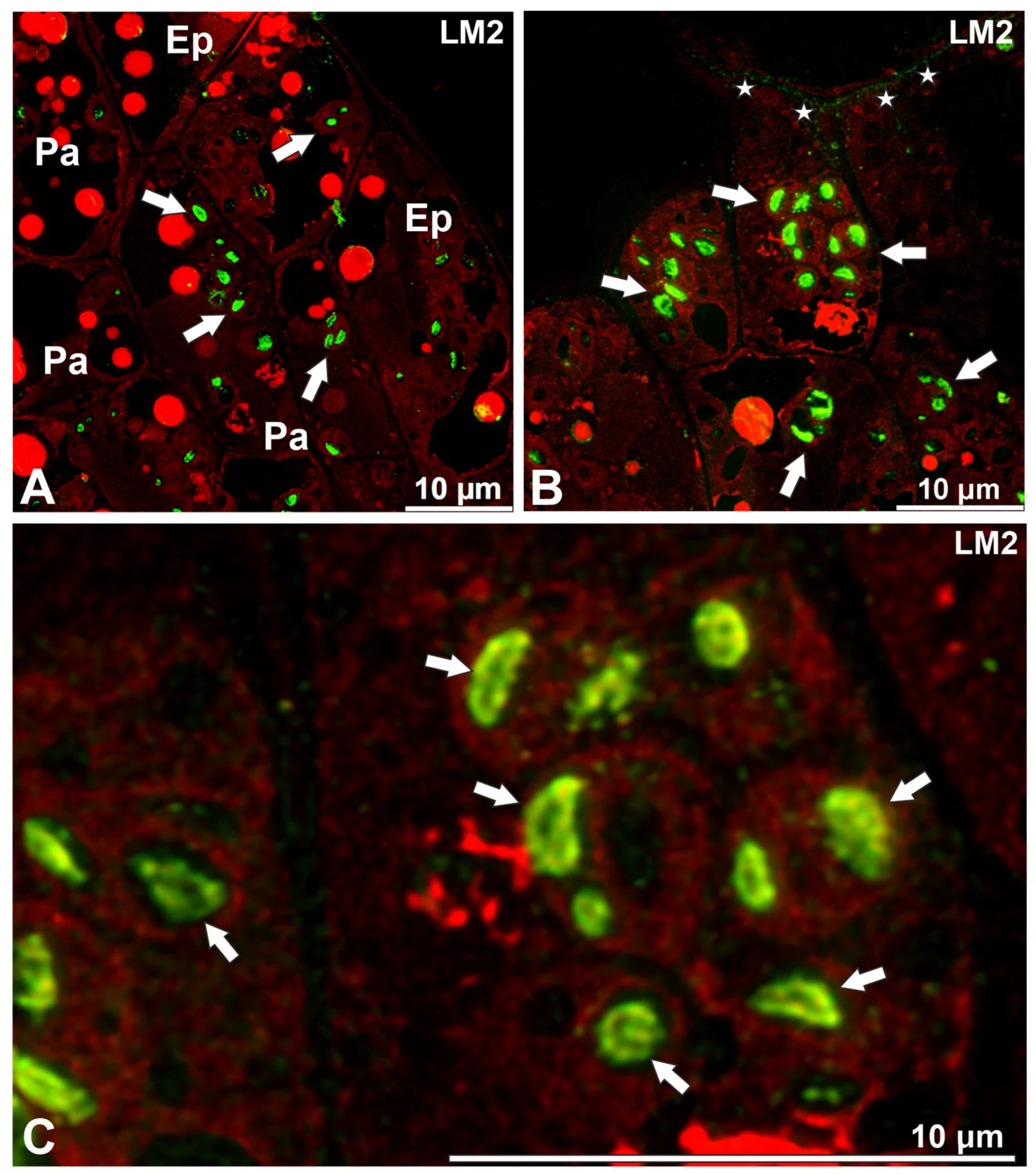
Distribution of AGP-reactive carbohydrate epitopes detected in the turion of *Aldrovanda vesiculosa* (intense green color—antibody-binding signal to the LM2 epitope; red–brown color—autofluorescence) by confocal microscopy. (**A**) Occurrence of LM2 epitopes in epidermal (Ep) and parenchyma (Pa) cells of the leaf. Note that LM2 epitopes occurred in starch grains (arrows). Bar, 10 μm. (**B**) Occurrence of LM2 epitopes in aerenchyma cells. Note that LM2 epitopes occurred in starch grains (arrows) and near cell walls (stars). Bar, 10 μm. (**C**) Greater enlargement of Figure B; LM2 epitopes occurred in starch grains (arrows). Bar, 10 µm.

**Figure 3:**
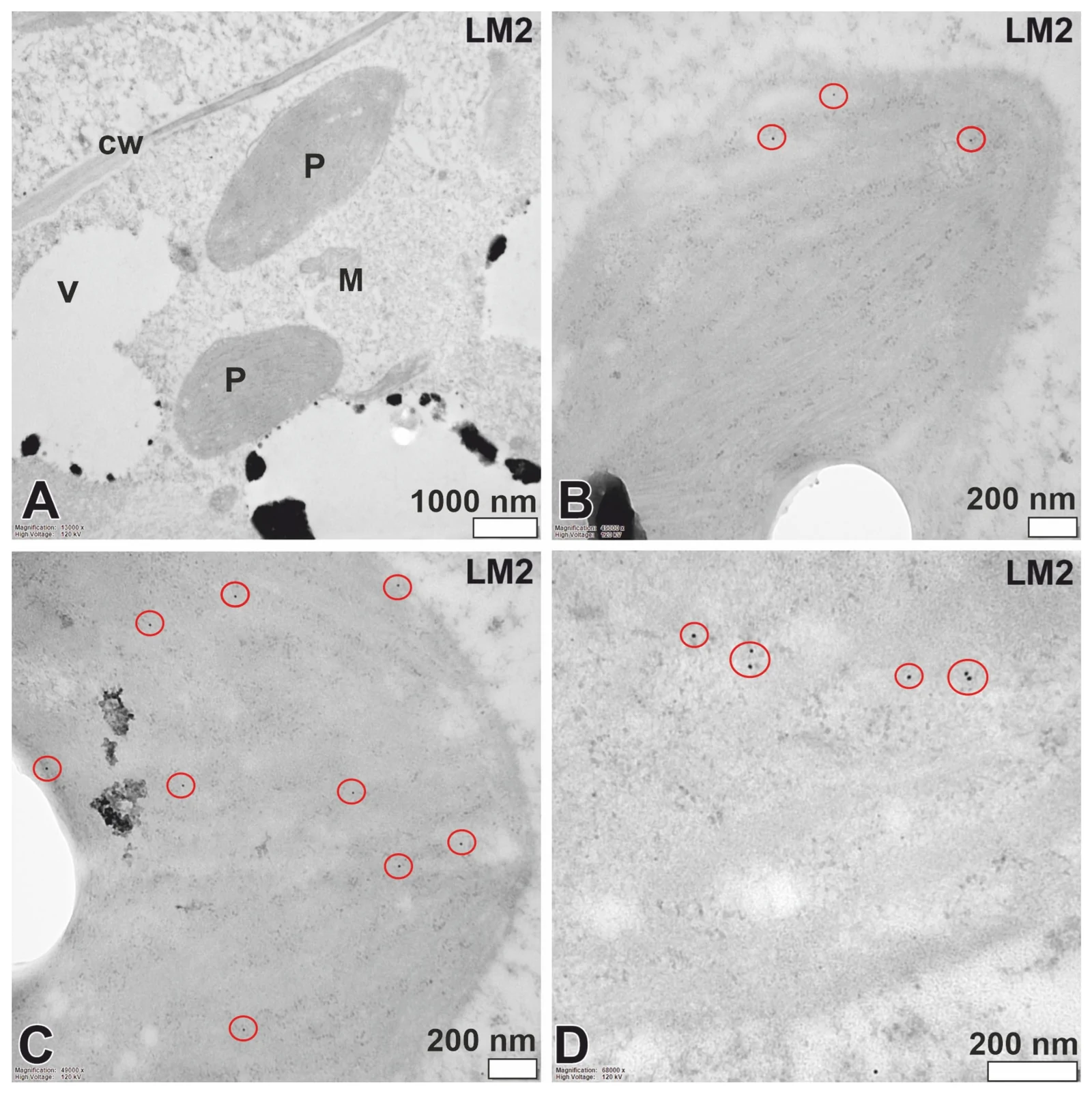
Immunogold labeling with LM2 in the plastids of leaf cells in the turion of *Aldrovanda vesiculosa*. (**A**) General ultrastructure. Plastids, P; mitochondrion, M; vacuole, V; cell wall, CW; bar, 1000 nm. (**B**–**D**) Immunogold labeling with LM2 in the plastids. Gold particles, red circles; bar, 200 nm.

**Figure 4:**
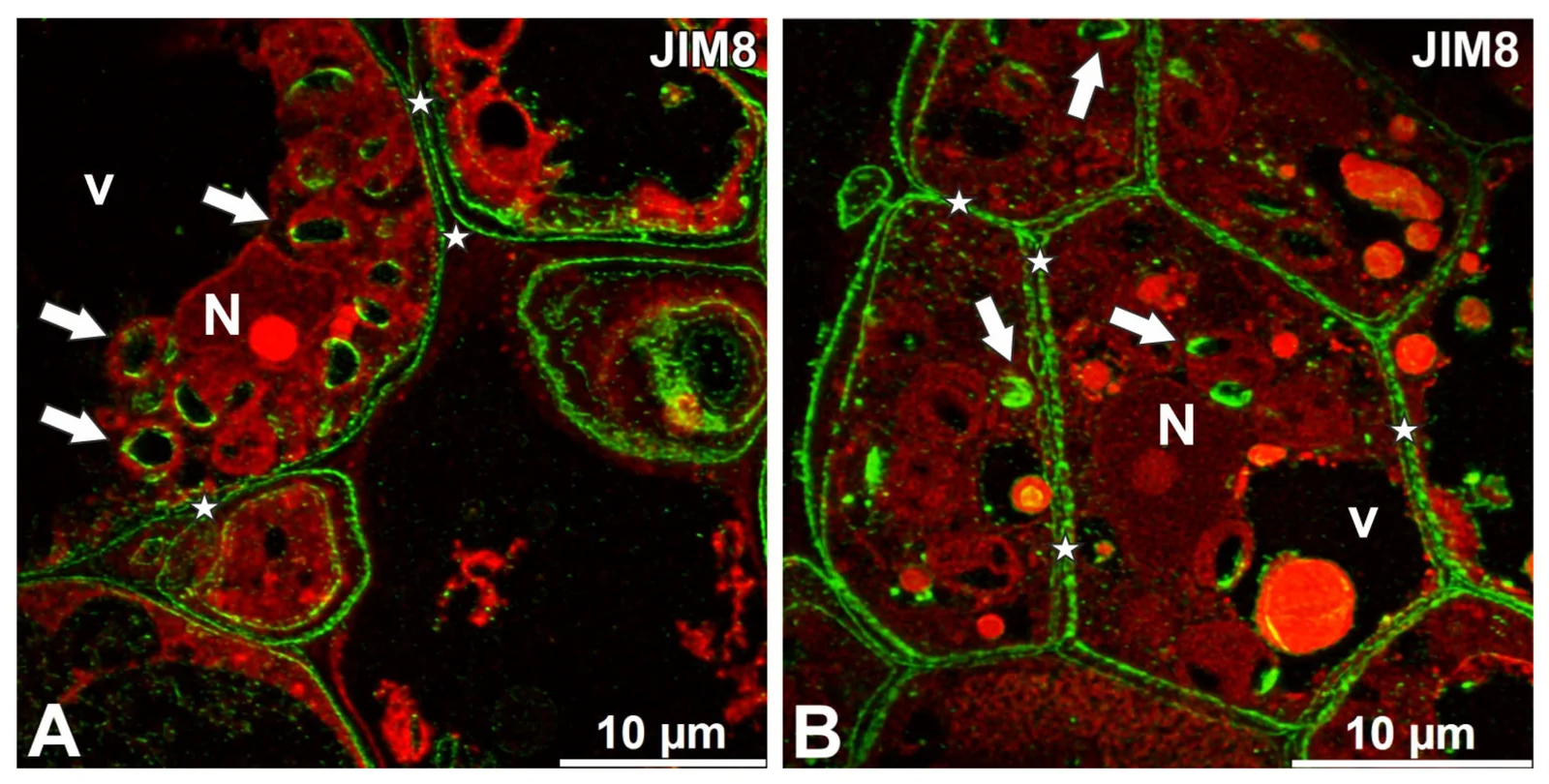
Distribution of AGP-reactive carbohydrate epitopes detected in the turion of *Aldrovanda vesiculosa* (intense green color—antibody-binding signal to the JIM8 epitope; red–brown color—autofluorescence) by confocal microscopy. (**A**,**B**) Occurrence of JIM8 epitopes in turion cells. Note that JIM8 epitopes occurred in starch grains (arrows) and near cell walls (stars). Vacuole, V; nucleus, N; bar, 10 μm.

**Figure 5:**
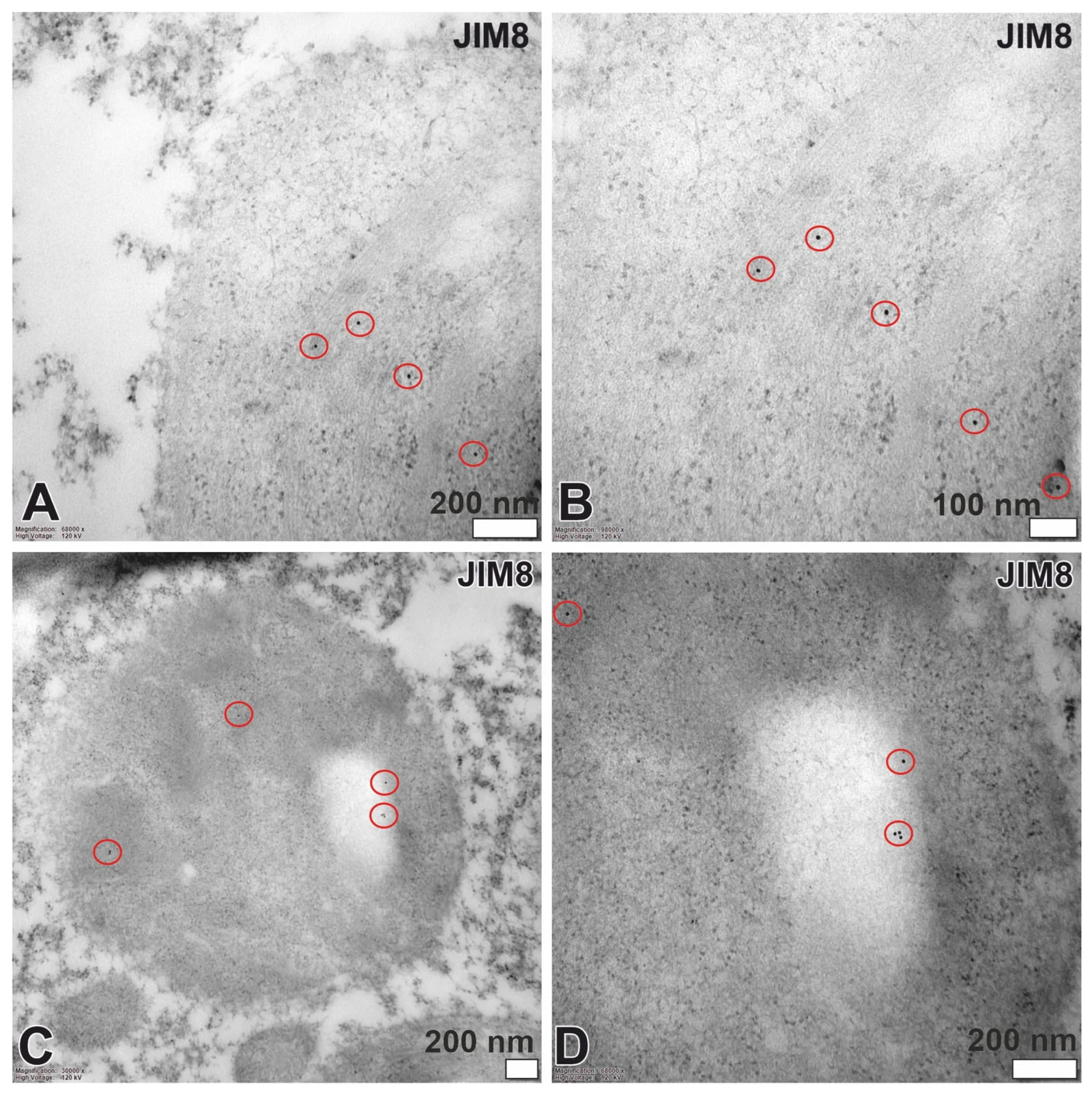
Immunogold labeling with JIM8 in the plastids of leaf cells in the turion of *Aldrovanda vesiculosa*. (**A**) Immunogold labeling with JIM8 in the chloroplast. Gold particles, red circles; bar, 200 nm. (**B**) The greater enlargement of Figure A. Bar, 200 nm. (**C**) Immunogold labeling with JIM8 in the plastid with starch. Gold particles, red circles. (**D**) The greater enlargement of Figure C. Bar, 200 nm.

## Data Availability

The original contributions presented in this study are included in the article/Supplementary Materials. Further inquiries can be directed to the corresponding author.

## Supplementary Materials

The following supporting information can be downloaded at: https://www.mdpi.com/article/10.3390/ijms27198452/s1, Figure S1: Control reactions of the immunolabeling of cell wall components. (A) Control reactions of cell wall components after immunolabeling (green color—signal of antibody, red-brown color—autofluorescence). Bar, 10 μm. (B) Immunogold labeling of cell wall components in turion cells. Bar, 200 nm.
