# Supplementary material for: Unexpected Localization of AGP Epitopes in the Plastids of Aldrovanda vesiculosa: New Questions About the Distribution of These Epitopes in Angiosperms
Source: Int J Mol Sci. 2026 Sep 22;27(19):8452. doi: 10.3390/ijms27198452 (PMC13665074; doi:10.3390/ijms27198452)

Figure S1

Figure S1. Control reactions of the immunolabeling of cell wall components. (**A**) Control reactions of cell wall components after immunolabeling (green color – signal of antibody, red-brown color—autofluorescence). Bar, 10 µm. (**B**) Immunogold labeling of cell wall components in turion cells. Bar, 200 nm.

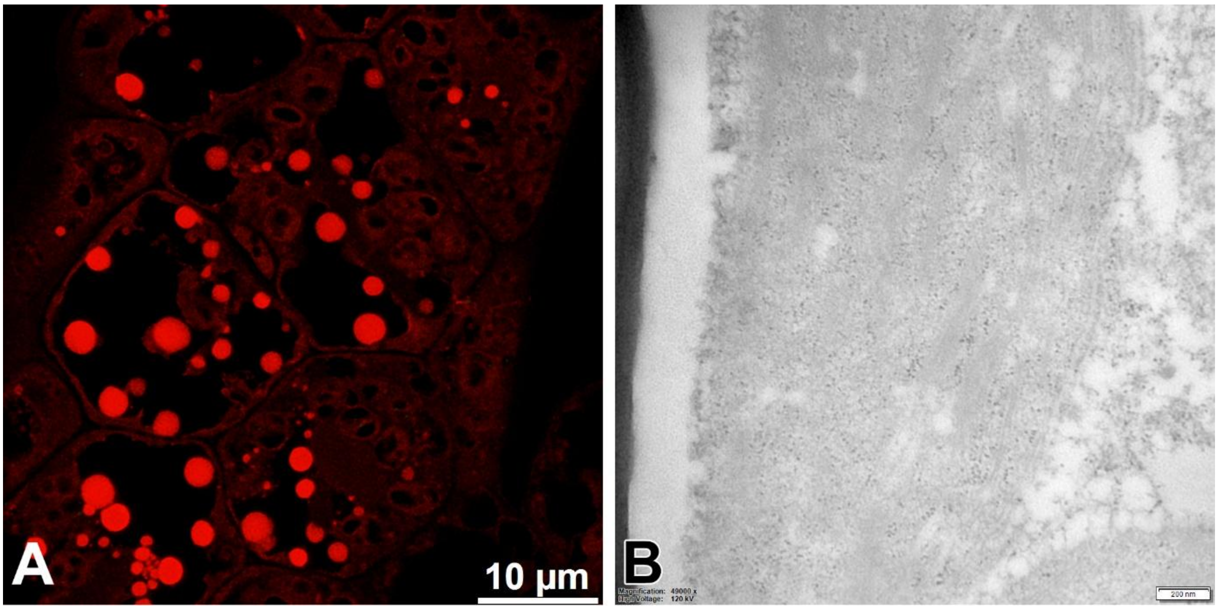

Supplement: Supplementary file 1 [file ijms-27-08452-s001.zip › ijms-4551469-supplementary.pdf]
